# Physics-Informed Neural Networks in Polymers: A Review

**DOI:** 10.3390/polym17081108

**Published:** 2025-04-19

**Authors:** Ivan Malashin, Vadim Tynchenko, Andrei Gantimurov, Vladimir Nelyub, Aleksei Borodulin

**Affiliations:** 1 Artificial Intelligence Technology Scientific and Education Center, Bauman Moscow State Technical University, 105005 Moscow, Russia; 2 Scientific Department, Far Eastern Federal University, 690922 Vladivostok, Russia

**Keywords:** physics-informed neural networks (PINNs), polymer modeling, multi-scale simulation, ML in materials science, structure–property relationships

## Abstract

The modeling and simulation of polymer systems present unique challenges due to their intrinsic complexity and multi-scale behavior. Traditional computational methods, while effective, often struggle to balance accuracy with computational efficiency, especially when bridging the atomistic to macroscopic scales. Recently, physics-informed neural networks (PINNs) have emerged as a promising tool that integrates data-driven learning with the governing physical laws of the system. This review discusses the development and application of PINNs in the context of polymer science. It summarizes the recent advances, outlines the key methodologies, and analyzes the benefits and limitations of using PINNs for polymer property prediction, structural design, and process optimization. Finally, it identifies the current challenges and future research directions to further leverage PINNs for advanced polymer modeling.

## 1. Introduction

Polymers exhibit complex behaviors due to their hierarchical structures, multi-scale interactions, and dependence on various environmental factors (such as temperature, pressure, and chemical composition). Traditional modeling approaches, such as molecular dynamics (MD) [1,2,3], Monte Carlo simulations [4,5,6], and continuum methods [7,8,9], although successful in capturing specific aspects of polymer behavior, face significant limitations. These approaches often struggle with scalability when dealing with large systems or long time scales as they require immense computational resources. Additionally, they face challenges in efficiently modeling multi-scale phenomena, such as the transition from molecular dynamics to macroscopic behavior.

Furthermore, traditional models often require substantial experimental data to ensure their accuracy and validation, and they may not readily adapt to new, unexplored material systems without extensive recalibration. For example, MD simulations are often confined to small system sizes and short time scales, while continuum models may oversimplify the complexities of polymer microstructures, ignoring molecular interactions.

In light of these challenges, physics-informed neural networks (PINNs) present a promising alternative. By combining machine learning (ML) with physics-based modeling, PINNs allow the incorporation of physical laws into the neural network architecture, ensuring that the model adheres to known scientific principles while learning from data. PINNs are effective in overcoming scalability issues by learning global patterns from data without the need for exhaustive pointwise simulations [10]. Moreover, PINNs can leverage optimization techniques that simultaneously minimize both the data-driven error and the residuals of the governing equations, thus offering a more accurate and computationally feasible approach to polymer modeling [11].

This paper aims to address these gaps by proposing a hybrid model that enhances the scalability and efficiency of traditional approaches while maintaining the rigor of physics-based constraints, ultimately leading to more accurate predictions of polymer behaviors across multiple scales.

**Figure 1:**
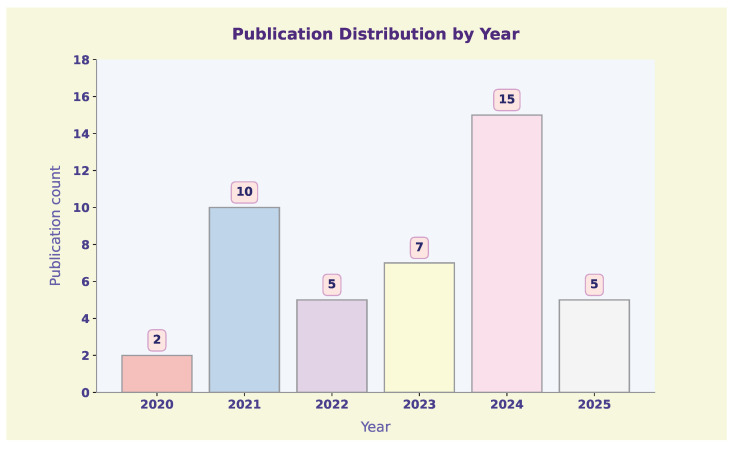
Year-wise distribution of PINN-based publications regarding polymers.

The number of publications on PINNs applied to polymers has shown a clear upward trend in recent years (Figure 1). From 2020 to 2022, the research activity remained relatively low, with only a few publications each year (two in 2020, ten in 2021, and five in 2022). However, starting in 2023, there was a noticeable increase, with the number of publications reaching seven, followed by a sharp rise to fifteen in 2024. This growth suggests a growing interest in using PINNs for polymer-related problems, likely driven by advancements in ML and increased recognition of the method’s potential in modeling complex polymer behaviors. While the data for 2025 are still emerging, with five publications recorded so far, the overall trend suggests a growing use of PINNs in polymer science.

**Figure 2:**
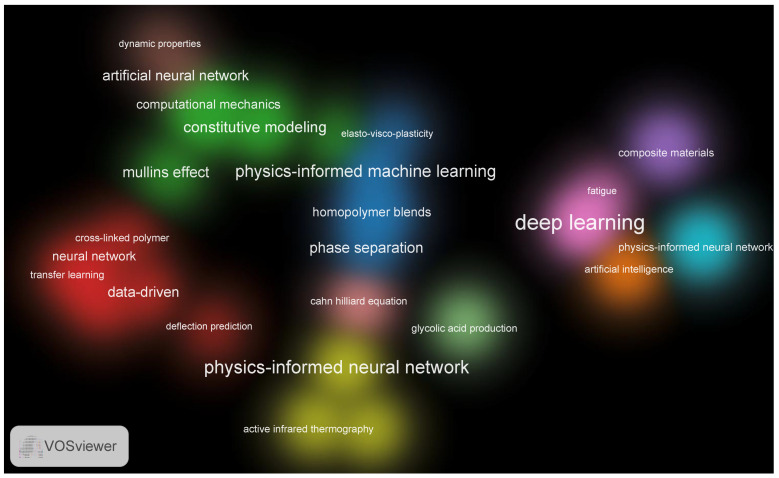
Keyword co-occurrence cluster map based on VOSviewer analysis.

The analysis of the keyword density provides insights into the key research directions and trends in the application of PINNs in polymer science (Figure 2). The VOSviewer-based visualization of keywords reveals a high concentration of terms related to ML, computational mechanics, and polymer property modeling. The most frequently occurring terms can be categorized into several main groups.

First, there are ML- and neural-network-based methods, including PINNs, Artificial Intelligence (AI), Artificial Neural Networks (ANNs), and Deep Learning, which indicate a growing interest in leveraging neural-network-based approaches to model complex physical phenomena in polymers. The presence of transfer learning [12,13,14] suggests ongoing efforts to generalize PINN applications across different polymeric systems.

Second, the visualization highlights key concepts related to computational mechanics and modeling, such as computational mechanics, constitutive modeling, Elasto-Visco-Plasticity, and Deflection Prediction, confirming the application of PINNs in simulating the mechanical properties of polymers, including their viscoelastic and plastic characteristics [15,16,17].

The third major theme includes polymer-science- and physics-based models, with terms such as phase separation, Cross-Linked Polymers, Homopolymer Blends, and the Cahn–Hilliard Equation, emphasizing the role of PINNs in modeling microstructure evolution and self-assembly processes in polymeric materials. The high density of the term Cahn–Hilliard Equation suggests the frequent application of PINNs [18,19] in modeling phase separation dynamics within polymer blends.

Additionally, experimental techniques and hybrid approaches appear, including Active Infrared Thermography, Dynamic Properties, and Fatigue, highlighting the integration of PINNs with real-world experimental methods to enhance predictive accuracy and model validation [20,21].

The interpretation of keyword density visualization reveals that PINNs are becoming a crucial tool in polymer science by enabling accurate physics-driven modeling while requiring minimal experimental data. The high concentration of physics-related terms, such as constitutive equations and phase behavior, indicates that PINNs are widely used for solving mechanical and thermodynamic problems in polymer systems.

Furthermore, the integration of PINNs with experimental methods like infrared thermography suggests a promising direction toward combining computational predictions with real-world measurements. This trend highlights the transition toward hybrid modeling approaches, where PINNs bridge the gap between data-driven learning and fundamental physics laws to create more accurate and interpretable models for polymer materials.

Future research directions may include expanding the use of PINNs for multi-scale modeling, enhancing model interpretability through uncertainty quantification, and integrating real-time experimental data to refine polymer models adaptively [22].

In addition to the well-established PINNs, several alternative physics-informed learning frameworks have recently emerged, aiming to address limitations in scalability, flexibility, and generalization. One such approach is the physics-informed neural operator (PINO) [23,24,25], which learns a mapping between entire functions rather than pointwise values. This operator-based formulation allows the PINO to generalize across different boundary conditions and material parameters, offering improved efficiency for high-dimensional or history-dependent systems.

Another notable method is the Physics-Embedded Neural Network (PENN) [26,27,28], which incorporates governing equations directly into the neural network architecture. Unlike PINNs that rely on residual-based losses, PENNs enforce physical constraints structurally, leading to potentially more stable and interpretable models.

Physics-guided machine learning (PGML) [29,30,31] represents a broader class of hybrid models where physical principles guide the model selection, feature engineering, or loss formulation. PGML is particularly useful in data-scarce scenarios as it leverages prior knowledge to reduce the solution space.

More recently, the concept of *super-constrained machine learning with L-agents* [32,33,34] has been introduced to enforce not only physical laws but also logic-based and multi-agent constraints within the learning process. L-agents can be used to represent various types of domain knowledge, including symbolic rules or empirical relations, allowing for highly structured and constrained learning suitable for complex engineering systems.

These emerging approaches complement the capabilities of PINNs and expand the toolkit available for solving inverse problems, multi-scale modeling, and uncertainty quantification in physics-driven domains.

This review examines the applications of PINNs in polymer science, focusing on their role in addressing complex problems that traditional computational methods encounter. The advantages of PINNs are analyzed, including their capacity to integrate physical laws into ML frameworks, leading to improved accuracy, data efficiency, and generalizability across various polymer systems. The methodologies employed in PINN-based modeling are discussed, covering areas such as constitutive modeling, degradation prediction, and process optimization. Some emerging challenges are identified, including computational cost, the necessity of high-quality experimental data, and limitations in capturing highly nonlinear behaviors in polymer systems. Potential future directions are considered, with an emphasis on hybrid modeling approaches, uncertainty quantification, and experimental validation to advance the field.

## 2. Theoretical Background on PINNs

PINNs [35] provide a framework for solving partial differential equations (PDEs) by embedding physical laws into the loss function of a neural network [36]. This method enables the approximation of solutions to PDEs without the need for extensive labeled data.

**Figure 3:**
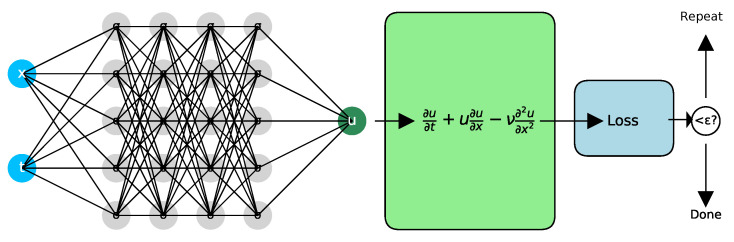
Schematic representation of PINN solving the Burgers equation.

Figure 3 illustrates the schematic structure of a PINN designed to solve a problem governed by the Burgers equation. The input to the neural network consists of spatial and temporal coordinates (x,t), which pass through several hidden layers with nonlinear activation functions σ. The network outputs the function *u*, which is then used to compute temporal and spatial derivatives. These derivatives are substituted into the governing PDE $\frac{\partial u}{\partial t}+u\frac{\partial u}{\partial x}-\nu \frac{\partial^{2}u}{\partial x^{2}}=0,$ where u=u(x,t) represents the solution, and ν is a given parameter controlling the viscosity. A loss function evaluates how well the network’s output satisfies the PDE. If the error exceeds a predefined tolerance ε, the neural network parameters are updated through gradient descent. This iterative process continues until the solution meets the specified accuracy criterion.

Let the governing equation for a polymer system be given by a general partial differential equation (PDE):

(1) N(u(x,t))=f(x,t),x∈Ω,t∈[0,T],

where N is a differential operator that encapsulates the underlying physics of the system, such as conservation laws, diffusion equations, or viscoelasticity models. The function u(x,t) represents the unknown solution to be approximated, which may describe properties like stress, strain, or concentration in the polymer system. The variable x denotes the spatial coordinates within the domain Ω, which can be one-, two-, or three-dimensional, while t∈[0,T] represents the time variable over a given interval. The term f(x,t) accounts for external forcing effects, such as applied loads, heat sources, or other external influences on the system.

The loss function for PINN consists of multiple components that enforce both data fidelity and physical consistency [37]:

(2) $$L=L_{\mathrm{data}}+\lambda L_{\mathrm{physics}}+\mu L_{\mathrm{BC}},$$

where L is the total loss function minimized during training. The term $L_{\mathrm{data}}$ represents the data loss, ensuring that the model predictions align with experimental or simulated observations. The term $L_{\mathrm{physics}}$ enforces the governing PDE constraints, ensuring that the neural network satisfies the differential equation N(u(x,t))=f(x,t). The boundary condition loss, $L_{\mathrm{BC}}$, enforces the required physical constraints at the domain boundaries Ω, such as fixed displacement or zero-flux conditions. The parameters λ and μ are weighting factors that balance the contributions of the physics and boundary loss terms relative to the data loss, allowing for better stability and accuracy in model training.

The data loss is defined as [38]

(3) $$L_{\mathrm{data}}=\sum_{i=1}^{N_{d}}{\left|u_{\mathrm{NN}}\left(x_{i},t_{i}\right)-u_{\mathrm{true}}\left(x_{i},t_{i}\right)\right|}^{2},$$

where $u_{\mathrm{NN}}\left(x,t\right)$ is the neural network approximation of the solution, and $u_{\mathrm{true}}\left(x,t\right)$ represents available observational data.

The physics-based loss term ensures the neural network adheres to the PDE constraints [39]:

(4) $$L_{\mathrm{physics}}=\sum_{j=1}^{N_{p}}{\left|N\left(u_{\mathrm{NN}}\left(x_{j},t_{j}\right)\right)-f\left(x_{j},t_{j}\right)\right|}^{2}.$$

This term penalizes deviations from the governing equations at a set of collocation points ${\left\{\left(x_{j},t_{j}\right)\right\}}_{j=1}^{N_{p}}$.

The boundary and initial conditions are incorporated through an additional constraint:

(5) $$L_{\mathrm{BC}}=\sum_{k=1}^{N_{b}}{\left|u_{\mathrm{NN}}\left(x_{k},0\right)-g\left(x_{k}\right)\right|}^{2}+\sum_{m=1}^{N_{c}}{\left|B\left(u_{\mathrm{NN}}\left(x_{m},t_{m}\right)\right)-h\left(x_{m},t_{m}\right)\right|}^{2}.$$

Here, B represents a boundary condition operator, and h(x,t) is the prescribed boundary value function.

To train the PINN, the total loss L is minimized using gradient-based optimization methods. Two widely used approaches include the following:

- Adam Optimizer: A first-order gradient-based method that adapts learning rates based on first and second moments of gradients, ensuring stable convergence.
- L-BFGS: A quasi-Newton method that often achieves faster convergence for smooth loss landscapes by leveraging second-order derivative approximations.

The optimization process aims to update the neural network parameters θ by computing the gradient of L [40]:

(6) $$\theta^{\left(k+1\right)}=\theta^{\left(k\right)}-\eta \nabla_{\theta }L,$$

where η is the learning rate. In the case of L-BFGS, an approximation to the inverse Hessian matrix $H_{k}$ is used as follows:

(7) $$\theta^{\left(k+1\right)}=\theta^{\left(k\right)}-H_{k}\nabla_{\theta }L.$$

The gradient $\nabla_{\theta }L$ is computed using automatic differentiation (AD), ensuring accurate and efficient backpropagation of errors. Training continues until convergence criteria, such as a tolerance on L or a maximum number of iterations, are met.

The gradient of L with respect to the neural network parameters θ is computed via automatic differentiation (AD), which provides an efficient way to obtain exact derivatives for complex nested functions [41]. The training process iteratively updates θ to minimize the total loss, thereby ensuring the learned solution satisfies both the data constraints and the governing physics.

PINNs offer several advantages over traditional numerical methods, including the following:

- Mesh-free formulation, allowing flexibility in handling complex geometries.
- The ability to incorporate sparse and noisy observational data.
- Implicit satisfaction of PDE constraints, reducing the need for explicit discretization.

**Figure 4:**
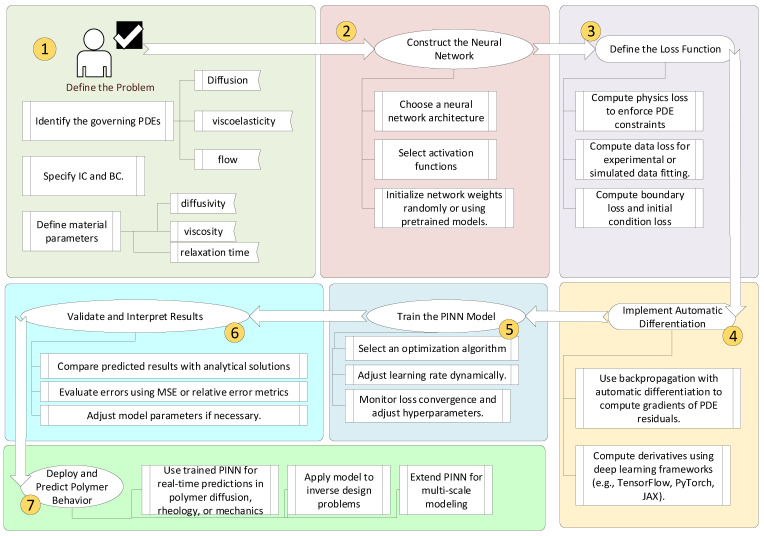
PINN pipeline.

Figure 4 is a flowchart that outlines the key steps in developing and applying a PINN framework for polymer science, from defining the governing equations to training the model and making predictions. Each step incorporates both computational and physical constraints, ensuring reliable and interpretable results.

## 3. Applications of PINNs in Polymer Science

### 3.1. Temperature

The temperature distribution during the curing process significantly affects the final quality of thermosetting composites. Ensuring temperature histories conform to specifications requires cure optimization, which fundamentally involves solving parametric coupled PDEs with dynamic boundary conditions [42]. Mathematically, this is expressed as solving

(8) $$\frac{\partial T}{\partial t}=k_{x}\frac{\partial^{2}T}{\partial x^{2}}+k_{y}\frac{\partial^{2}T}{\partial y^{2}}+k_{z}\frac{\partial^{2}T}{\partial z^{2}}+\dot{Q}$$

where *T* represents the temperature of the polymer system, which varies with spatial position and time. The parameters $k_{x},k_{y},k_{z}$ denote the thermal conductivities in the *x*-, *y*- and *z*-directions, respectively, capturing the anisotropic heat transfer properties of the material.

The nonlinear exothermic heat source, $\dot{Q}$, accounts for the heat generated within the system due to chemical reactions or phase transformations. It is given by

(9) $$\dot{Q}=v_{r}\rho_{r}H_{r}\frac{\partial \alpha }{\partial t}.$$

In this equation, $v_{r}$ represents the reaction rate, which governs the speed of the exothermic process. The parameter $\rho_{r}$ denotes the density of the reacting species, influencing the overall energy release. The term $H_{r}$ is the reaction enthalpy, quantifying the amount of heat generated per unit mass of reactant. Finally, $\frac{\partial \alpha }{\partial t}$ represents the time derivative of the reaction progress variable α, which describes the extent of the reaction occurring in the system. As α evolves over time, the heat generation rate $\dot{Q}$ dynamically changes, affecting the overall temperature distribution.

Recently, PINNs have emerged as promising solvers for PDEs without labeled data [43]. Conventional PINNs approximate solutions in a pointwise manner, requiring a large number of collocation points, leading to a computational burden of $O(N^{3})$ for training. Instead, Meng et el. [23] proposed a physics-informed neural operator (PINO) approach, mapping the entire cure cycle to temperature and degree of cure (DoC) histories as a function-to-function operator for carbon-fiber-reinforced polymers [44] (CFRP) composites. By enforcing global constraints on field outputs, the PINO achieves unsupervised parametric PDE solving while reducing training complexity to O(NlogN) using Fourier neural operators (FNOs).

Compared to traditional PINNs, the PINO introduces a fundamental shift in the learning paradigm. While PINNs aim to approximate the solution u(x,t) by minimizing the residuals of governing PDEs (e.g., L[u]=0) at discrete collocation points, PINOs learn an operator that maps entire input functions to output solution functions. Specifically, the PINO seeks a functional mapping

G:f(x,t)↦u(x,t),

where f(x,t) represents inputs such as boundary/initial conditions or material parameters, and u(x,t) denotes the solution field (e.g., temperature or degree of cure).

In the context of cure modeling for carbon-fiber-reinforced polymers, the PINO maps the full cure cycle to temperature and degree of cure (DoC) histories across space and time. Unlike PINNs, which compute physics residuals pointwise and often encounter scalability issues in high-dimensional problems, the PINO leverages neural operator architectures (e.g., Fourier neural operators) to generalize across varying input conditions with reduced computational cost.

PINNs enforce physical laws explicitly by including PDE residuals in the loss function:

$$L_{\mathrm{physics}}={∥N\left[u_{\theta }\right]\left(x,t\right)∥}^{2},$$

whereas the PINO captures the physics implicitly through training on solution data that satisfy the underlying equations. This makes the PINO suitable for rapid predictions in parametric or history-dependent systems. To highlight the key methodological differences between PINN and PINO, Table 1 provides a side-by-side comparison of their core features, strengths, and suitable application scenarios.

The PINO model reduces training time compared to the fully connected physics-informed neural network (FC-PINN), a standard PINN architecture composed of fully connected layers. In FC-PINN, the network approximates mappings from inputs (e.g., spatial and temporal coordinates) to outputs (e.g., temperature or degree of cure), with physical laws enforced via PDE residuals in the loss function. While FC-PINN serves as a baseline architecture, the PINO achieves much faster convergence, requiring only 84.16 s for a 260-min one-dwell cure cycle, compared to 2370 s for for FC-PINN, while achieving a lower temperature MAE of 0.2 K compared to 1.6 K. For two-dwell and smart cure cases, the PINO maintains high accuracy with temperature MAE values of 0.273 K and 0.257 K, respectively, and DoC MAE values below 0.007. The parametric study shows that, with 50 training samples, the PINO achieves temperature MAE of 0.267 ± 0.068 K for one-dwell and 0.226 ± 0.039 K for two-dwell cases, with relative percentage errors below 0.08%. Training time increases for parametric cases, reaching 3016.61 s for one-dwell and 4880.85 s for two-dwell, yet inference remains highly efficient. The resolution-invariance of the PINO is confirmed as training at a lower resolution (*▵t* = 8 s) still provides accurate predictions at a higher resolution (▵t=4s) while reducing computational cost.

While the PINO improves computational efficiency and accuracy in solving parametric PDEs for cure optimization, it still faces notable limitations. The model requires substantial training time for complex parametric cases, reaching 4880.85 s for two-dwell curing, which may limit real-time industrial applications. Despite its improved generalization, the PINO’s accuracy depends on the quality and distribution of training samples, making it sensitive to under-represented regions in the parametric space. Additionally, the reliance on FNO may introduce limitations in capturing highly localized temperature gradients, particularly in heterogeneous composites [45]. Finally, while the PINO reduces computational complexity to O(NlogN), achieving further scalability for high-dimensional and multiphysics problems remains an open challenge.

### 3.2. Viscosity

The production of high-quality polymeric components through additive manufacturing (AM) relies on precise control of melt viscosity (η), which depends on molecular weight ($M_{w}$), shear rate ($\dot{\gamma }$), and temperature (*T*). The viscosity follows a shear-thinning behavior, modeled by

(10) $$\eta \left(M_{w},T,\dot{\gamma }\right)=\frac{\eta_{0}\left(M_{w},T\right)}{1+{\left(\frac{\dot{\gamma }}{{\dot{\gamma }}_{cr}}\right)}^{1-n}}$$

where $\eta_{0}\left(M_{w},T\right)$ represents the zero-shear viscosity, ${\dot{\gamma }}_{cr}$ is the critical shear rate, and *n* describes shear sensitivity. The temperature dependence is captured by the WLF equation:

(11) $$\eta_{0}=\eta_{M_{w}}\times 10^{-\frac{C_{1}\left(T-T_{r}\right)}{C_{2}+\left(T-T_{r}\right)}}$$

where $C_{1}$ and $C_{2}$ are empirical parameters, and $T_{r}$ is a reference temperature near the glass transition temperature. Molecular weight dependence follows a piecewise power law:

(12) $$\eta_{M_{w}}=\left\{\begin{array}{cc} k_{1}M_{w}^{\alpha_{1}}, & M_{w}<M_{cr}\\ k_{2}M_{w}^{\alpha_{2}}, & M_{w}\geq M_{cr} \end{array}\right.$$

where $M_{cr}$ is the critical molecular weight, with typical values $\alpha_{1}\approx 1$ and $\alpha_{2}\approx 3.4$. This formulation reflects distinct viscosity behaviors in different molecular weight regimes:

- When $M_{w}<M_{cr}$: The polymer chains are relatively short, and viscosity follows a weak power law dependence with $\alpha_{1}\approx 1$. In this regime, the entanglement between polymer chains is minimal, resulting in a nearly linear increase in viscosity with increasing molecular weight.
- When $M_{w}>M_{cr}$: The polymer chains exceed the critical entanglement threshold, leading to a significant increase in viscosity characterized by $\alpha_{2}\approx 3.4$. This steep increase is attributed to the formation of an entangled polymer network, which restricts molecular motion and enhances resistance to flow.
- When $M_{w}=M_{cr}$: This represents the transition point where polymer viscosity shifts from the dilute or semi-dilute regime to the entangled regime. At this critical molecular weight, the polymer chains begin to overlap and form entanglements, drastically altering the rheological behavior.

To predict viscosity in unexplored domains, a Physics-Enforced Neural Network (PENN) was developed by Jain et al. [46], enforcing these dependencies while predicting parameters such as *n*, ${\dot{\gamma }}_{cr}$, and $C_{1},C_{2}$. The PENN outperforms physics-unaware ANN and GPR models in extrapolating η for unseen $M_{w}$, $\dot{\gamma }$, and *T*, improving predictive accuracy for novel polymers in AM applications.

The melt viscosity dataset consists of 1903 datapoints, including 1326 homopolymer, 446 co-polymer, and 113 miscible polymer blend samples, spanning 93 unique repeat units with variations in molecular weight (Mw), shear rate ($\dot{\gamma }$), temperature (T), and polydispersity index (PDI). To address under-representation of viscosity (η) at low Mw, 126 additional datapoints were extrapolated using empirical relationships. Model accuracy was assessed using Order of Magnitude Error (OME), with the Physics-Enforced Neural Network (PENN) improving OME by 35.97% on average and achieving up to 79% $R^{2}$ for $\dot{\gamma }$ predictions. Compared to Gaussian Process Regression (GPR) and Artificial Neural Network (ANN), the PENN demonstrated lower Kullback–Leibler divergence in empirical parameter estimation, with RMSE values of 0.05 for $\alpha_{1}$ and 0.17 for $\alpha_{2}$, closely matching theoretical values of 1 and 3.4. The PENN model also outperformed ANN and GPR in capturing shear thinning behavior, with predicted *n* values between 0.2 and 0.8 and shear rates (${\dot{\gamma }}_{cr}$) closely aligned with experimental distributions.

While the PENN model enhances viscosity prediction accuracy, its reliance on extrapolated data for low-molecular-weight regions introduces potential bias and uncertainty. The model’s performance is constrained by the availability of high-quality experimental data as errors may propagate when predicting viscosity for novel polymer chemistries with limited training samples [47]. Although the PENN improves OME by 35.97% and achieves up to 79% $R^{2}$, its accuracy in predicting extreme viscosity conditions (e.g., highly entangled polymer networks) remains unverified. The use of empirical constraints ensures physically plausible predictions, but it may limit the model’s flexibility in capturing unexpected behaviors in complex polymer systems. Finally, while the PENN outperforms ANN and GPR in shear-thinning modeling, further validation across broader AM process conditions is required to confirm its robustness in real-world applications.

### 3.3. Viscoelasticity

Physics-guided ML (PGML) methods integrate both data and physical knowledge, making them valuable for modeling the constitutive relations of solids. While PGML approaches have successfully modeled elasticity and plasticity, viscoelasticity remains a challenge due to its dependence on time and loading paths [48]. Many existing methods require extensive experimental or simulation data, making accurate modeling difficult in data-scarce scenarios. Qin et al. [49] proposed a physics-guided recurrent neural network (RNN) model combining gated recurrent units (GRUs) and feedforward neural networks (FNNs) to predict the viscoelastic behavior of solids. The model takes time and stretch (or strain) sequences as inputs, allowing stress predictions based on time and loading paths. A physics-guided initialization strategy using stress–stretch data from the generalized Maxwell model helps to mitigate data scarcity.

Consider a solid $B_{0}$ bounded by $\partial B_{0}$, which deforms into $B_{t}$ with surface $\partial B_{t}$. The deformation gradient is given by

(13) $$F=\nabla_{R}x$$

where x=χ(X,t) maps an arbitrary material point X in $B_{0}$ to a spatial point in $B_{t}$.

The deformation gradient F describes how an infinitesimal material element in the reference configuration $B_{0}$ deforms into the current configuration $B_{t}$. The correlation between the deformation gradient and the domain transformations is as follows:

- $B_{0}$ (Reference Configuration): This represents the undeformed or initial state of the body, where material points are labeled by their initial coordinates X. The deformation gradient F is computed relative to this configuration.
- $\partial B_{0}$ (Boundary of the Reference Configuration): This is the initial boundary of the material body before deformation. As deformation occurs, boundary points in $\partial B_{0}$ are mapped to new positions on $\partial B_{t}$, governed by F.
- $B_{t}$ (Current Configuration): This is the deformed state of the solid at time *t*. The transformation x=χ(X,t) determines the new position of every material point from $B_{0}$ to $B_{t}$. The tensor F quantifies the local stretch and rotation from $B_{0}$ to $B_{t}$.
- $\partial B_{t}$ (Boundary of the Current Configuration): The deformed boundary of the material body, which evolves from $\partial B_{0}$ under the transformation x=χ(X,t).

The deformation gradient F relates directly to the changes in surface elements of $B_{0}$ and $B_{t}$. Specifically, changes in the normal vectors and area elements of $\partial B_{0}$ and $\partial B_{t}$ can be expressed using F and its determinant, which represents local volume changes. The rate of deformation can be analyzed through its time derivative, often linked to velocity gradients in continuum mechanics. Thus, the deformation gradient provides a link between the reference and current configurations, enabling the analysis of strain, stress, and material behavior under deformation.

Using the generalized Maxwell model, the deformation gradient decomposes as

(14) $$F=F_{i}^{e}\cdot F_{i}^{v},\;i=1,\cdots ,n$$

where $F_{i}^{e}$ and $F_{i}^{v}$ are the elastic and viscous deformation gradients. The Cauchy stress tensor satisfies

(15) $$\sigma \cdot \nabla +b=0,\;\sigma =\sigma^{T}$$

which, in the reference configuration, takes the form:

(16) $$P\cdot \nabla_{R}+b_{R}=0,\;P\cdot F^{T}=F\cdot P^{T}$$

where P is the first Piola–Kirchhoff stress tensor. The energy balance equation is

(17) $$\dot{\varepsilon }=\sigma :\nabla \dot{\chi }-\nabla \cdot j_{q}+q$$

Introducing the Helmholtz free energy density $\phi_{R}=\varepsilon -\vartheta \eta$, the entropy inequality is

(18) $$\sigma :\nabla \dot{\chi }-\eta \dot{\vartheta }-{\dot{\phi }}_{R}-\frac{1}{\vartheta }j_{q}\cdot \nabla \vartheta \geq 0$$

Assuming the free energy function:

(19) $$\phi_{R}=\phi_{R}\left(C,C_{i}^{e},\vartheta \right)$$

the first Piola–Kirchhoff stress tensor is obtained as

(20) $$P=2F\cdot \frac{\partial \phi_{R}}{\partial C}+2F_{i}^{e}\cdot \frac{\partial \phi_{R}}{\partial C_{i}^{e}}\cdot {\left(F_{i}^{v}\right)}^{T}$$

For incompressible materials, the Cauchy stress is

(21) $$\sigma =\frac{G^{eq}L}{L-\mathrm{tr}C+3}F\cdot F^{T}+\sum_{i=1}^{n}\frac{G_{i}^{neq}L_{i}}{L_{i}-\mathrm{tr}C_{i}^{e}+3}F_{i}^{e}\cdot {\left(F_{i}^{e}\right)}^{T}-\left(\Pi +\sum_{i=1}^{n}\Pi_{i}\right)I$$

where $G^{eq}$ and $G_{i}^{neq}$ are equilibrium and non-equilibrium moduli, and Π enforces incompressibility. The relaxation time of the Maxwell elements is

(22) $$\tau_{i}=\frac{\nu_{i}}{G_{i}^{neq}}$$

where $\nu_{i}$ is viscosity. The generalized Maxwell model and PGML together enable accurate viscoelastic predictions with limited data.

To handle history-dependent behaviors in viscoelasticity prediction, the GRU-FNN [50] model is trained using Backpropagation Through Time (BPTT). Four datasets from stress–stretch experiments at stretching rates of 0.025/s, 0.05/s, 0.10/s, and 0.20/s on VHB4905 samples (130 mm × 10 mm × 0.5 mm) were split into training and testing sets. The model’s RMSE values at 313 K were 0.81, 1.32, 1.72, and 4.55, and, at 333 K, they were 0.24, 0.31, 0.53, and 4.27. The loss evolution across epochs showed improved generalization with additional training data. Sensitivity analysis indicated increased noise levels led to higher prediction errors, with RMSE values varying across datasets.

The PGML approach improves viscoelastic modeling but remains dependent on dataset quality and preprocessing choices. While the physics-guided initialization mitigates data scarcity, the model’s accuracy still relies on the availability of representative experimental data. The GRU-FNN architecture captures history-dependent behaviors, yet its performance varies significantly across different stretching rates and temperatures. High RMSE values at faster stretching rates indicate potential limitations in handling rapid mechanical responses. Further validation on diverse material classes and loading conditions is necessary to assess the model’s robustness beyond the tested dataset.

### 3.4. Inelasticity

Data-driven approaches in solid mechanics offer a new paradigm, overcoming traditional constitutive model limitations such as complexity and accuracy. However, challenges such as high-dimensional data, missing information, and limited convergence hinder machine-learning applications in material modeling. Ghaderi et al. [51] introduced a reduced-order framework by leveraging polymer science, statistical physics, and continuum mechanics to develop super-constrained machine-learning techniques. A sequential order-reduction approach simplifies the 3D stress–strain tensor mapping into 1D problems, classified systematically using multiple replicated neural network learning agents (L-agents). Each L-agent captures specific deformation behaviors using first and second deformation invariants:

(23) $$l1_{di}=\sqrt{diCdi},\;l2_{di}=\sqrt{diC^{-1}di},\;C=F^{T}F$$

For rubber inelasticity, 21 teams of 2 agents each are trained with deformation memory using $l_{j}^{di}$ parameters. The final cost function integrates fusion constraints and thermodynamic consistency:

(24) $$E\left(W1,W2\right)=\frac{1}{2}\sum_{n=1}^{N}{\left[g1\left(\sum_{i=1}^{21}\sum_{j=1}^{2}w_{i}\frac{\partial A_{i}^{j}}{\partial l_{j}^{di}}\frac{\partial l_{j}^{di}}{\partial F}-pF^{-T}\right)g1-P_{n}\right]}^{2}$$

To minimize training data requirements, confidence intervals are defined based on agent contributions, ensuring accurate predictions across different deformation states. Training strategies with uni-axial, bi-axial, and compression data demonstrate superior accuracy within confidence intervals. The model was trained using various datasets, including Mars, Treloar, and Heuillet, with uni-axial, bi-axial, and pure shear tests [52].

Training with bi-axial data only until c=1.65 resulted in limited confidence intervals for uni-axial and pure shear predictions, where variable *c* represents the training progress or iteration limit at which the model is trained using specific datasets, with the value of *c* indicating the extent of the data used for training in each stage.

When trained using uni-axial data until c=2.18, the model showed a confidence limit of c=2.18 in shear but only c=1.21 in bi-axial due to uncertainty in training L-agent 2.

Extending uni-axial training to c=7.7 increased the confidence interval for bi-axial to c=1.66, demonstrating that training length impacts predictive accuracy. A combined uni-axial tensile (c=3.7) and compression (c=0.4) dataset improved confidence in bi-axial predictions up to c=1.58 and pure shear up to c=3.7. The proposed model achieved a prediction error of 1.12% for Treloar’s dataset, outperforming WYPiWYG (5.26%) and the network averaging tube model (2.11%) while being significantly less data-dependent.

The reduced-order framework improves computational efficiency but relies on predefined constraints, which may limit flexibility for complex materials. While the sequential order-reduction approach simplifies high-dimensional stress–strain relationships, its effectiveness depends on the chosen invariants and deformation modes [53]. The confidence interval strategy helps to manage uncertainty, yet the model’s accuracy varies based on training data coverage. Discrepancies in bi-axial and shear predictions suggest sensitivity to specific loading conditions, requiring careful dataset selection. Further testing across diverse materials and loading histories is needed to evaluate its applicability beyond rubber inelasticity.

### 3.5. Aging

Ghaderi et al. [54] introduced a novel physics-informed multi-agent constitutive model for predicting the quasi-static constitutive behavior of cross-linked elastomers and their mechanical performance loss due to environmental aging. The model simulates single-mechanism chemical aging (i.e., thermal-induced or hydrolytic aging), which alters the polymer matrix over time due to chain scission, chain formation, and molecular rearrangement.

A data-driven super-constrained ML engine was developed to represent damage in the polymer matrix, capturing inelastic features such as the Mullins effect and permanent set during aging. The complex 3D stress–strain tensor mapping is reduced to a set of constrained 1D problems via sequential order reduction. A system of neural network learning agents (L-agents) were trained to simplify these mappings while ensuring thermodynamic consistency.

The constitutive model is built using multiple constraints:

- Strain-energy-based formulation: The strain energy function $\Psi_{m}$ is used as an intermediate variable in stress–strain mapping, ensuring material objectivity and thermodynamic consistency:
  (25) $$P=\frac{\partial \Psi_{m}}{\partial F},\;S=\frac{\partial \Psi_{m}}{\partial E},\;\tau =\frac{\partial \Psi_{m}}{\partial L}.$$
- 3D-to-1D transition using a microsphere model: The polymer matrix is represented as a network of 1D elements distributed on a unit sphere, where the strain energy is obtained via numerical integration:
  (26) $$\Psi_{m}\approx \sum_{i=1}^{N_{d}}w_{i}\Psi_{m}^{d_{i}},$$
  where $\Psi_{m}^{d_{i}}$ is the energy contribution of an element in direction $d_{i}$.
- Network decomposition: The polymer matrix is divided into parallel networks, each describing a specific inelastic effect, leading to a superposition formulation:
  (27) $$\Psi_{m}=\sum_{i=1}^{N_{d}}\sum_{j=1}^{N_{s}}w_{i}\Psi_{j}^{d_{i}}.$$

The first Piola–Kirchhoff stress tensor P is then derived as

(28) $$P=\sum_{i=1}^{N_{d}}\sum_{j=1}^{N_{s}}w_{i}\frac{\partial A_{j}^{d_{i}}}{\partial F}-pF^{-T},$$

where *p* is the Lagrange multiplier ensuring incompressibility.

A conditional neural network (CondNN) [55] architecture is used for the L-agents, incorporating both mechanical and environmental damage. The network consists of two branches: one capturing mechanical damage and the other representing environmental effects, combined multiplicatively to model aging effects accurately.

The model introduces constraints to ensure thermodynamic consistency, but these restrictions may limit adaptability to materials with complex aging mechanisms. The microsphere-based 3D-to-1D transition reduces computational cost, yet the accuracy depends on the chosen discretization and weighting scheme. While network decomposition captures multiple inelastic effects, its predictive performance relies on well-calibrated L-agents, which may require extensive training data [56]. The conditional neural network approach accounts for environmental aging, but interactions between mechanical and chemical degradation might not be fully captured. Further validation across diverse elastomer formulations and exposure conditions is necessary to assess its broader applicability.

### 3.6. Deflection

To characterize the highly nonlinear state response of the Ionic Polymer–Metal Composite (IPMC), coupled with inherent response uncertainties, Zhang et al. [57] reformulated differential equations that encapsulate the highly nonlinear deflection of IPMC, accounting for uncertainties, and proposed a data-driven approach utilizing a physics-informed neural network (PINN) to effectively solve this differential equation and predict the nonlinear deflection of IPMC actuators.

The electric field induces cation migration, and the relationship between the local voltage and ion charge can be described by the Poisson equation:

(29) $$\nabla^{2}\phi =-\frac{\rho }{k_{e}}=-\frac{F(C^{+}-C^{-})}{k_{e}}$$

where ϕ is the electric potential, ρ is the charge density, $k_{e}$ is the effective dielectric constant of the polymer, *F* is the Faraday constant, and $C^{+}$ and $C^{-}$ are the concentrations of cations and anions, respectively.

The continuity expression relates the ion flux to the cation concentration:

(30) $$\nabla \cdot J=-\frac{\partial C^{+}}{\partial t}$$

where *J* is the ion flux, which consists of diffusion, migration, and convection components:

(31) $$J=-d\left(\nabla C^{+}+\frac{C^{+}F}{RT}\nabla \phi +\frac{C^{+}\Delta V}{RT}\nabla p\right)+C^{+}v$$

where *d* is the diffusion coefficient, *R* is the gas constant, ΔV is the volumetric change, *T* is the absolute temperature, *p* is the pressure, and *v* is the free water velocity field.

Neglecting nonlinear terms, the partial differential equation for charge density is

(32) $$\frac{\partial \rho }{\partial t}-d\frac{\partial^{2}\rho }{\partial x^{2}}+\frac{F^{2}dC^{-}}{k_{e}RT}\left(1-C^{-}\Delta V\right)\rho =0$$

The nonlinear control partial differential equation for the IPMC electric field is

(33) $$\frac{\partial^{2}E}{\partial t\partial x}=d\left(\frac{\partial^{3}E}{\partial x^{3}}-\frac{F(1-C^{-}\Delta V)}{RT}\left[\frac{\partial^{2}E}{\partial x^{2}}E+{\left(\frac{\partial E}{\partial x}\right)}^{2}\right]-\frac{F^{2}C^{-}\left(1-C^{-}\Delta V\right)}{RTk_{e}}\frac{\partial E}{\partial x}\right)$$

For nonlinear deflection of an IPMC actuator, the geometric deformation relationship is given by

(34) $$\frac{dw}{ds}=\sin\theta ,\;\frac{dx}{ds}=\cos\theta ,\;\frac{dw}{dx}=\tan\theta$$

(35) $$\frac{d\theta }{ds}=\frac{1}{r},\;\frac{d^{2}\theta }{ds^{2}}+\frac{U}{B}\sin\theta =0$$

where *U* is the input voltage, and *B* is the bending stiffness of the IPMC.

A PINN integrates information from both measurement results and the governing partial differential equations into the loss function:

(36) $$MSE=MSE_{u}+MSE_{f}$$

where

(37) $$MSE_{u}=\frac{1}{N}\sum_{i=1}^{N_{u}}{\left|u\left(t_{u}^{i},x_{u}^{i}\right)-u_{i}\right|}^{2},\;MSE_{f}=\frac{1}{N_{f}}\sum_{i=1}^{N_{f}}{\left|f(t_{f}^{i},x_{f}^{i})\right|}^{2}$$

ensuring accurate modeling of the system.

The experimental results show that Kolmogorov–Arnold Neural PINN (KAN-PINN) [58,59] achieves a 27.54% improvement in prediction accuracy compared to Multilayer-Perceptron-based PINN (MLP-PINN) [60], with an average relative error of 0.316% for Pt-IPMC and 0.277% for Ag-IPMC. KAN-PINN also reduces the convergence cycle significantly, reaching convergence at Epoch 500 for Pt-IPMC and Epoch 400 for Ag-IPMC, compared to Epoch 1400 and Epoch 1200 for MLP-PINN, respectively. However, KAN-PINN’s per-iteration training time is approximately ten times longer than MLP-PINN, leading to a longer total training time. The total loss of KAN-PINN is 10.3% lower for Pt-IPMC and 25.75% lower for Ag-IPMC, demonstrating superior convergence. Ag-IPMC exhibits higher voltage sensitivity and lower surface resistance, resulting in faster convergence and improved accuracy compared to Pt-IPMC.

The PINN-based approach leverages physical laws to guide learning, but its accuracy depends on the proper formulation of differential equations and boundary conditions. The method reduces data dependence, yet the increased computational cost, especially with KAN-PINN, may limit practical applications. While KAN-PINN achieves lower loss and faster convergence per epoch, its per-iteration training time is significantly higher than MLP-PINN, leading to longer total training times. The model’s performance varies between Pt-IPMC and Ag-IPMC, suggesting material-dependent factors influence prediction accuracy and convergence rates. Further validation across diverse IPMC compositions and operating conditions is needed to assess its robustness in real-world applications.

### 3.7. Polymerization

Multiphysics engineering impacts chemical reactors due to the complex interactions among fluid mechanics, chemical reactions, and transport phenomena, which significantly impact reactor performance. Recently, PINNs have been successfully applied to engineering problems due to their ability to generalize across domains. Ryu et al. [61] introduced a novel application of PINNs for modeling multiphysics in a chemical reactor. They examined the effectiveness of PINNs in reconstructing and extrapolating ethylene conversion in a polymerization reactor. CFD simulations were conducted to generate training data, and a PINN model was built by incorporating conventional neural network loss with the residuals of fundamental physics equations: continuity, Navier–Stokes [62], and species transport.

The governing equations for fluid flow include the Reynolds-averaged Navier–Stokes equation:

(38) $$\frac{d(\rho v)}{dt}=-\nabla \cdot \left(\rho vv\right)-\nabla P+\mu \nabla^{2}v+\tau_{Re}$$

and the continuity equation:

(39) $$\frac{d\rho }{dt}=-\nabla \cdot \left(\rho v\right).$$

The reactor was modeled as incompressible with constant density and viscosity given its operation at low conversion ( 20%). The rotating motion of the stirrer was approximated using a moving reference frame, leading to the modified Navier–Stokes equation for relative velocity $v_{r}$:

(40) $$v_{r}=v-\Omega \times r$$

(41) $$\frac{d(\rho v_{r})}{dt}=-\nabla \cdot \left(\rho v_{r}v_{r}\right)-\nabla P+\mu \nabla^{2}v_{r}+\tau_{Re}+\Omega \times \left(\Omega \times r\right)+2\Omega \times v_{r}.$$

Radical polymerization kinetics were described using three primary reactions: initiator decomposition, polymerization, and termination. The kinetic laws are

(42) $$I\to 2\lambda ,\;k_{d}=1.54\times 10^{14}\exp\left(\frac{-15023}{T}\right)$$

(43) $$M+\lambda \to P,\;k_{p}=1.25\times 10^{8}\exp\left(\frac{-3800}{T}\right)$$

(44) $$\lambda +\lambda \to P,\;k_{t}=1.25\times 10^{9}\exp\left(\frac{-327}{T}\right).$$

PINN training involved minimizing a loss function composed of empirical loss and physical residuals:

(45) $$L=w_{emp}\frac{1}{N_{emp}}\sum_{i=1}^{N_{emp}}|y_{i}^{\mathrm{approx}}-y_{i}^{\mathrm{CFD}}{|}^{2}+\frac{1}{N_{phy}}\sum_{i=1}^{N_{phy}}{\left|L_{i}^{\mathrm{physical}}\right|}^{2}.$$

The physical residuals incorporated continuity, Navier–Stokes, and species balance equations:

(46) $$L_{i}^{\mathrm{physical}}=w_{\mathrm{cont}}L_{i}^{\mathrm{cont}}+w_{\mathrm{NS}}L_{i}^{\mathrm{NS}}+w_{\mathrm{species}}L_{i}^{\mathrm{species}}.$$

The results demonstrated that PINNs accurately predicted ethylene concentration profiles with an 18% lower mean absolute error (0.1028 mol/L) compared to conventional neural networks (0.1267 mol/L). Furthermore, PINNs successfully captured the conversion concaveness effect, a unique feature in radical polymerization, whereas traditional neural networks failed to do so. These findings highlight the potential of PINNs to efficiently model and extrapolate multiphysics in chemical reactors.

The use of PINNs allows the model to incorporate physical laws, reducing reliance on extensive CFD training data. However, the accuracy of PINN predictions depends on the proper weighting of empirical and physics-based loss terms, which can be challenging to tune. While the model outperforms conventional neural networks in capturing ethylene concentration profiles, its effectiveness in highly turbulent or non-ideal reactor conditions remains uncertain. The approach assumes constant density and viscosity, which may limit its applicability to systems with significant property variations. Further studies are needed to assess PINN robustness when applied to different reactor designs and operating conditions.

### 3.8. Rheology

Time- and rate-dependent material functions in non-Newtonian fluids pose challenges in integrating constitutive models into CFD. The goal is to solve coupled PDEs relating shear stress to deformation, capturing fluid behavior under different conditions. Mahmoudabadbozchelou et al. [63] introduce non-Newtonian physics-informed neural networks (nn-PINNs) to solve these PDEs using automatic differentiation (AD), eliminating the need for mesh generation.

The power law (PL) model, representing shear-thinning or shear-thickening behavior, is

(47) $$\tau_{xy}=\eta {\dot{\gamma }}^{n}$$

where η is the consistency index and *n* is the power law exponent.

The Carreau–Yasuda (CY) model accounts for viscosity variation with shear rate:

(48) $$\tau_{xy}=\eta_{\infty }+\left(\eta_{0}-\eta_{\infty }\right){\left[1+{\left(\lambda \dot{\gamma }\right)}^{a}\right]}^{(n-1)/a}\dot{\gamma }$$

where λ, *a*, and *n* define viscosity transition characteristics.

Yield stress fluids are modeled using the Bingham plastic model:

(49) $$\tau_{xy}=\tau_{y}+\eta \dot{\gamma }$$

where $\tau_{y}$ is the yield stress.

The Herschel–Bulkley (HB) model generalizes yield stress fluids:

(50) $$\tau_{xy}=\tau_{y}+\eta {\dot{\gamma }}^{n}$$

The Maxwell model incorporates viscoelastic effects:

(51) $$\tau_{xy}+\frac{\eta }{G}{\dot{\tau }}_{xy}=\eta \dot{\gamma }$$

where *G* is the elastic modulus.

For more complex behavior, the TEVP model includes elastic, plastic, and thixotropic effects:

(52) $$\left\{\begin{array}{c} {\dot{\tau }}_{xy}\left(t\right)=G\frac{1}{\eta_{s}+\eta_{p}}\left[-\tau_{xy}\left(t\right)+\tau_{y}l\left(t\right)+\left(\eta_{s}+\eta_{p}l\left(t\right)\right)\dot{\gamma }\left(t\right)\right]\\ \dot{l}\left(t\right)=k_{+}\left(1-l\left(t\right)\right)-k_{-}l\left(t\right)\dot{\gamma }\left(t\right) \end{array}\right.$$

where $k_{+}$ and $k_{-}$ define structure evolution.

The governing equations for incompressible fluid motion are

(53) ∇·v=0

(54) $$\frac{Dv}{Dt}=-\frac{1}{\rho }\nabla p+\frac{1}{\rho }\nabla \cdot \tau$$

where v is velocity, *p* is pressure, and ρ is density.

Loss functions in nn-PINN training minimize residuals from PDEs and boundary conditions:

(55) $$\mathrm{MSE}={\mathrm{MSE}}_{R}+w_{2}{\mathrm{MSE}}_{ICs}+w_{3}{\mathrm{MSE}}_{BCs}$$

where each term represents the squared error in the residuals.

nn-PINN framework successfully predicts the spatiotemporal behavior of NN fluids with a maximum error of 4% for power law fluids and under 2% for generalized Newtonian fluid (GNF) models. The model accurately captures transitions between shear-thinning (n = 0.8), Newtonian (n = 1.0), and shear-thickening (n = 1.2) behaviors while maintaining consistency across different flow protocols. In complex cases, such as viscoelastic and thixotropic models, nn-PINN reconstructs velocity and stress fields with as few as 50 sparse data points. The framework also adapts to unknown boundary conditions, including slip effects, demonstrating its robustness. Overall, nn-PINN generalizes well across a range of constitutive equations, providing an efficient and reliable alternative for solving fluid dynamics problems.

The nn-PINN framework eliminates the need for mesh generation, but its reliance on automatic differentiation can lead to high computational costs for large-scale simulations. While it accurately captures shear-dependent behaviors, its performance for highly nonlinear viscoelastic fluids with strong memory effects remains uncertain. The approach assumes well-defined constitutive models, which may limit its ability to handle fluids with poorly understood or evolving properties [64]. Despite its ability to adapt to unknown boundary conditions, the sensitivity of its predictions to noise in sparse data points needs further investigation. Future work should explore its applicability to real-world industrial flows where multiple nonlinear effects interact simultaneously.

## 4. Future Perspectives

Various physics-informed ML approaches have been proposed for modeling polymeric and composite materials. Table 2 provide a comparative analysis of the studied methods based on key characteristics, including the mathematical formulation, data requirements, prediction accuracy, computational efficiency, novelty of the approach, and its limitations.

The integration of PIML into polymeric and composite material modeling has demonstrated significant potential in improving predictive accuracy, reducing computational costs, and enhancing generalization capabilities. However, several challenges and opportunities remain for future research.

One major direction is the development of interpretable models that can account for complex physical interactions while maintaining computational efficiency. The current PIML models often require extensive hyperparameter tuning and struggle with extrapolation beyond training data. Future advancements in PIML may focus on adaptive learning techniques and hybrid models that adjust based on real-time experimental feedback [65]. Adaptive learning methods can integrate data such as temperature, pressure, or molecular weight distributions, allowing models to update predictions during processes like polymerization or extrusion [66]. Active learning may help to select informative data points when data are limited, reducing the need for large datasets. Online learning techniques enable models to adjust parameters as new data are received, supporting real-time process control in polymer processing [67]. Hybrid models combine physics-based models with data-driven approaches by incorporating physical laws and neural network methods to capture nonlinear relationships in polymer behavior [68]. Merging equations for heat transfer, fluid flow, and rheology with data-driven techniques provides predictions of polymer properties under varying conditions. The integration of adaptive learning with hybrid models allows PIML methods to update predictions as new data become available while managing uncertainty [69]. This approach may enhance the design, processing, and performance prediction of polymers in various industries.

Another area is data scarcity and uncertainty quantification [69,70]. Many polymeric and composite systems suffer from limited experimental datasets, making it challenging to train data-driven models effectively. Techniques such as transfer learning, active learning, and Bayesian inference can be leveraged to improve model reliability under sparse-data conditions. Furthermore, incorporating physics-based uncertainty quantification methods will be essential for increasing confidence in model predictions, particularly in safety-critical applications such as aerospace and biomedical engineering.

Advancements in multiphysics and multi-scale modeling present another promising research avenue. The current approaches primarily focus on either macroscale mechanical properties or microscale material behavior, but future research should aim to bridge these scales seamlessly. Coupling PIML with molecular dynamics (MD), density functional theory (DFT), and continuum mechanics could enable more comprehensive material characterization.

Additionally, real-time and edge computing applications for physics-informed models [71,72] could revolutionize industrial processes. Deploying lightweight PIML models on embedded systems and IoT devices could enable in situ monitoring of material behavior during manufacturing, leading to enhanced quality control and predictive maintenance strategies.

Finally, benchmarking and standardization of PIML methodologies are methods for broader adoption. Establishing open-source datasets, evaluation metrics, and standardized training protocols will help to compare different approaches objectively and accelerate their integration into industry workflows.

By addressing these challenges and opportunities, future research can unlock the full potential of physics-informed ML, paving the way for more accurate, efficient, and generalizable models in polymer and composite material science.

## 5. Further Applications of PINNs in Polymers

Physics-informed neural networks (PINNs) have shown great promise in a wide range of applications beyond those discussed in this paper.

PINNs can be employed to optimize polymer processing techniques, such as extrusion, injection molding, and 3D printing. By embedding the physical laws governing heat transfer, fluid flow, and mechanical behavior into the neural network, PINNs can predict the temperature distribution, viscosity, and stress fields in real time, improving process control and product quality [73]. Additionally, PINNs can be used to optimize the design of molds and predict the behavior of polymers during the curing or cooling stages. For example, in recent works, PINNs have been applied to improve the manufacturing processes of polymer-based bone scaffolds, optimizing heat distribution and processing parameters in 3D printing [74].

PINNs can be used to model complex rheological behaviors in polymer melts and solutions, particularly in scenarios involving non-Newtonian fluids or shear-thinning/thickening behavior. By incorporating constitutive models like the Carreau–Yasuda or Bingham models into PINNs, researchers can predict viscosity and flow stress under varying shear rates, temperatures, and pressure conditions [75]. This is useful in simulating flow in confined spaces or through complex geometries, such as during polymer extrusion or in microfluidic devices. As demonstrated in recent studies, PINNs have been applied to simulate polymer rheology and improve predictions of composite material properties [76,77].

The degradation of polymers over time, due to environmental factors such as temperature, UV radiation, and mechanical stress, can also be modeled using PINNs. PINNs can be trained to capture the physical chemistry of polymer degradation, such as the breakage of chemical bonds, chain scission, and the formation of new cross-links [78]. This application is valuable for predicting the long-term performance and lifetime of polymers in various industries, including the packaging, automotive, and biomedical sectors. In one recent study, PINNs were used to predict the accelerated creep behavior of polymer-alloy geocell sheets, simulating aging effects under different conditions [79].

PINNs can be applied to the modeling of polymer blends and composites, where multiple phases and interactions between different polymer components exist. By embedding the governing equations for phase separation, diffusion, and material property changes in the neural network, PINNs can predict the properties of these complex materials under different processing conditions [80]. This could be particularly beneficial in optimizing the properties of high-performance materials for aerospace, automotive, or electronics applications. Recent work on polymer nanocomposites has utilized PINNs for more accurate predictions of material properties [81,82].

The behavior of polymer nanocomposites, which involve the incorporation of nanoparticles to enhance mechanical, thermal, and electrical properties, can also be predicted using PINNs. These materials often exhibit complex interactions between the polymer matrix and the nanoparticles, and PINNs can be used to model and predict how these interactions influence macroscopic properties such as stiffness, strength, and electrical conductivity [83]. Additionally, PINNs can be applied to the study of smart polymers that respond to external stimuli like temperature, pH, or electric fields, enabling the development of adaptive and responsive materials. Recent studies have demonstrated the potential of PINNs to predict the behavior of such advanced materials [84].

PINNs can be used to predict the molecular weight distribution (MWD) of polymer systems during polymerization processes. This is crucial for controlling the material properties of the polymer, such as tensile strength, elasticity, and melting temperature. By embedding the kinetic equations for polymerization reactions and incorporating experimental data, PINNs can provide insights into how changes in reaction parameters [85] (e.g., temperature, pressure, and initiator concentration) influence the MWD and, consequently, the material properties. Recent advancements in this area include the use of PINNs to predict polymer molecular weight and rheological behavior [81,86].

In future research, it could be beneficial to combine this study with other recent advancements in related fields, such as optical neural ODEs [87], deep neural networks for phase hologram generation, recurrent neural networks in laser modeling, and photonic neural network acceleration. These approaches may provide further insights and broaden the scope of PIML applications

The application of holographic flow equations and neural ODEs to polymers, in strongly coupled systems such as viscoelastic fluids, can be formulated as the shear response of a polymer in a strongly coupled system being governed by a modified flow equation [88]:

(56) $$\frac{\partial }{\partial z}\chi +i\omega \frac{f(z)}{z^{2}}\left(\chi^{2}-\frac{1}{z^{2}}\right)-\frac{1}{i\omega }\frac{m^{2}}{z^{4}}=0,$$

where χ represents the polymer shear response, f(z) is a function related to the polymer network’s elasticity, and $m^{2}$ is associated with the interactions between polymer chains.

The real part of the shear viscosity $\eta_{\mathrm{re}}$, which quantifies the polymer’s response to shear stress [89], is given by

(57) $$\eta_{\mathrm{re}}=\chi_{\mathrm{re}}{|}_{z\to 0}.$$

This relationship reflects how the polymer’s internal structure, such as entanglements and elasticity, influences its macroscopic flow behavior.

The dynamics of polymer flow are described by an ODE, which can be solved numerically using a neural ODE framework. The shear response of the polymer is governed by the following equations:

(58) $$\frac{d\chi_{\mathrm{re}}}{dz}=2\omega z^{2}f\chi_{\mathrm{re}}\chi_{\mathrm{im}},$$

(59) $$\frac{d\chi_{\mathrm{im}}}{dz}=\frac{\omega }{f}\left({\left(z\chi_{\mathrm{im}}\right)}^{2}-{\left(z\chi_{\mathrm{re}}\right)}^{2}+\frac{1}{z^{2}}-\frac{m^{2}}{\omega z^{4}}\right).$$

In these equations, $\chi_{\mathrm{re}}$ and $\chi_{\mathrm{im}}$ represent the real and imaginary components of the shear response, and f(z) describes the polymer’s elasticity. The mass term $m^{2}$ represents internal entropic forces within the polymer.

The function f(z) (which governs the polymer’s elasticity) or the mass term $m^{2}\left(z\right)$ can be parameterized by a neural network with trainable parameters θ:

(60) $$\frac{dx(t)}{dt}=y\left(x\left(t\right),t,\theta \right).$$

This network learns how the shear response of the polymer evolves over time or distance, enabling accurate modeling of the polymer’s viscoelastic behavior.

After training, the shear viscosity of the polymer at high frequencies (in the UV limit) can be extracted as follows:

(61) $$\eta_{\mathrm{re}}=\chi_{\mathrm{re}}\left(z=0\right).$$

It provides a quantitative understanding of the polymer’s flow properties, making it possible to predict the material’s response to various conditions.

These applications highlight the potential of PINNs in polymer science and engineering. By integrating governing physical laws with experimental data, PINNs offer a useful approach for addressing problems where conventional modeling methods may face limitations, particularly in the context of complex multiphysics interactions and sparse data. As the field advances, PINNs have the potential to contribute to the design, processing, and performance prediction of polymers across various industries.

## 6. Conclusions

PINNs present a transformative approach to polymer modeling by embedding governing equations directly into the learning process. This review has discussed their applications, advantages, and challenges. While hurdles remain, advances in computational strategies and model architectures are expected to further enhance their impact in polymer science. The main findings can be summarized as follows:

- PIML bridges the gap between data-driven and physics-based modeling, enabling more accurate and interpretable predictions in polymeric and composite materials.
- The integration of domain knowledge enhances model reliability and generalization, ensuring thermodynamic consistency and reducing dependency on large datasets.
- Computational efficiency remains a critical trade-off, with advanced models achieving high accuracy but requiring significant training time and computational resources.
- Multiphysics and multi-scale modeling are key to capturing complex material behaviors, allowing for better predictions in nonlinear, time-dependent, and high-dimensional problems.
- Extrapolation and uncertainty quantification remain challenges as some models struggle with under-represented data regimes and noise sensitivity.
- Standardization and benchmarking across studies are necessary to establish best practices and facilitate industrial adoption of PIML approaches.
- Future advancements should focus on hybrid models, adaptive learning strategies, and real-time deployment, paving the way for predictive material design and intelligent manufacturing.
- Computational efficiency varies across models, with the training times ranging from 84.16s for simple cases to 3016.61 s for parametric studies, demonstrating trade-offs between accuracy and complexity.
- Data requirements remain a challenge, with models trained on datasets ranging from 50 sparse experimental points to 1903 viscosity data points, highlighting the need for improved data efficiency.
- Extrapolation and noise sensitivity limit generalizability, with some models achieving up to 79% *R*^2^ accuracy, but others show prediction errors of up to 5.26% in challenging deformation states.
- Multiphysics and multi-scale approaches enhance performance, with PINN-based models reducing mean absolute error (MAE) by 18% compared to conventional neural networks in polymerization modeling.
- Thermodynamic consistency and physics constraints improve reliability, with constrained ML approaches reducing prediction error to as low as 1.12%, outperforming purely data-driven models.
- Standardization and benchmarking are essential as variability in performance metrics (RMSE ranging from 0.24 to 4.55) complicates direct comparisons across studies.
- Industrial adoption remains an ongoing challenge, but real-time deployment could enable in situ monitoring and predictive maintenance, leading to improved material processing and manufacturing efficiency.

## Figures and Tables

**Table 1:** Comparison between PINN and PINO.

Feature	PINN	PINO
Learning Target	Pointwise function approximation	Operator (function-to-function mapping)
Input/Output	Scalar coordinates → scalar solution	Function → function
PDE Enforcement	Explicit via loss function	Implicit through training data or regularization
Scalability	Moderate (can be slow for complex PDEs)	High (efficient once trained)
Generalization	Limited to trained domain	Strong across varying inputs
Suitable Use Case	Low- to moderate-dimensional PDE solutions	High-dimensional, parametric PDE problems

**Table 2:** Comparison of physics-informed models across different studies.

Aspect	Meng et al. [23]	Jain et al. [46]	Qin et al. [49]	Ghaderi et al. [51]
Key Challenge	Optimizing temperature distribution during curing	Predicting viscosity for AM polymers	Modeling viscoelasticity under time-dependent loads	Overcoming high dimensionality in stress–strain modeling
Model Proposed	PINO (Physics-Informed Neural Operator)	PENN (Physics-Enforced Neural Network)	PGML (Physics-Guided RNN with GRU-FNN)	Super-constrained ML with L-agents
Mathematical Formulation	Solves parametric coupled PDEs with dynamic BCs	Shear-thinning viscosity models (WLF equation)	Generalized Maxwell model for stress–strain prediction	Reduced-order representation with first and second deformation invariants
Data Used	50 training samples for parametric study	1903 viscosity data points (homopolymers, co-polymers, blends)	Stress–stretch data at different strain rates (VHB4905)	Uni-axial, bi-axial, and shear test datasets (Mars, Treloar, Heuillet)
Key Performance Metrics	MAE: 0.2–0.273 K (temperature), 0.007 (DoC)	35.97% improvement in OME, RMSE: 0.05 ($\alpha_{1}$), 0.17 ($\alpha_{2}$), $R^{2}$ up to 79%	RMSE at 313K: 0.81–4.55; RMSE at 333K: 0.24–4.27	Prediction error: 1.12% (Treloar), outperforming WYPiWYG (5.26%)
Computational Efficiency	Training time: 84.16s (1-dwell), 3016.61s (parametric)	More efficient than ANN and GPR	Uses Backpropagation Through Time (BPTT) for efficiency	Order reduction improves efficiency in high-dimensional problems
Novelty and Advantages	Function-to-function mapping reduces training complexity to O(NlogN)	Captures viscosity trends with physics-aware constraints	Combines data-driven and physics-based learning for better generalization	Reduces ML dependency on extensive datasets while ensuring thermodynamic consistency
Limitations	Increased training time for parametric cases	Requires extrapolation for under-represented viscosity regions	Noise sensitivity affects prediction accuracy	Limited confidence intervals in some deformation states
**Aspect**	**Ghaderi et al. [54]**	**Zhang et al. [57]**	**Ryu et al. [61]**	**Mahmoudabadbozchelou et al. [63]**
Key Challenge	Predicting mechanical performance loss in aging elastomers	High nonlinearities and response uncertainties in IPMC bending	Coupling of fluid mechanics, chemical reactions, and transport phenomena	Complex constitutive equations, varying flow conditions
Model Proposed	Multi-agent constitutive model with neural network learning agents (L-agents)	Physics-Informed Neural Network (PINN) for solving nonlinear PDEs	PINN-based ethylene conversion model for radical polymerization reactor	nn-PINNs
Mathematical Formulation	Strain-energy-based formulation, microsphere model, network decomposition	Poisson equation, charge transport PDEs, nonlinear beam deflection equations	Navier–Stokes equations, continuity equation, radical polymerization kinetics	Power law, Carreau–Yasuda, Herschel–Bulkley, Maxwell, and TEVP models
Data Used	Simulated aging dataset	Experimental IPMC deflection data	CFD-simulated reactor data	Sparse experimental and simulated data (50 sparse points)
Key Performance Metrics	Captures Mullins effect and permanent set	27.54% improved accuracy over MLP-PINN, lower error rates (0.316%, 0.277%)	18% lower mean absolute error compared to conventional NN (0.1028 vs. 0.1267 mol/L)	Maximum error 4% (power law), under 2% for generalized Newtonian fluids
Computational Efficiency	3D stress–strain mapping reduced to constrained 1D problems	Faster convergence, but higher per-iteration training time	Efficiently models multiphysics interactions, capturing conversion concaveness	Adapts to unknown boundary conditions, eliminates need for meshing
Novelty and Advantages	Ensures thermodynamic consistency via constrained ML models	Captures electromechanical coupling and improves generalization of PINN models	Successfully reconstructs and extrapolates polymerization profiles where traditional ML fails	Generalizes across diverse constitutive models, effective in sparse-data regimes
Limitations	Requires extensive hyperparameter tuning for stability	Sensitive to parameter initialization and requires extensive labeled data	Computationally expensive for highly nonlinear coupled systems	Struggles with extreme flow conditions and requires careful scaling for different regimes

## References

[1] Hollingsworth S.A., Dror R.O. (2018). Molecular dynamics simulation for all. Neuron.

[2] Tang Y., Fu Z., Raos G., Ma F., Zhao P., Hou Y. (2024). Molecular dynamics simulation of adhesion at the asphalt-aggregate interface: A review. Surf. Interfaces.

[3] Li Y., Chen R., Zhou B., Dong Y., Liu D. (2024). Rational design of DNA hydrogels based on molecular dynamics of polymers. Adv. Mater..

[4] Kalateh F., Kheiry M. (2024). A review of stochastic analysis of the seepage through earth dams with a focus on the application of monte carlo simulation. Arch. Comput. Methods Eng..

[5] Schiavo M. (2024). Numerical impact of variable volumes of Monte Carlo simulations of heterogeneous conductivity fields in groundwater flow models. J. Hydrol..

[6] Gawusu S., Ahmed A. (2024). Analyzing variability in urban energy poverty: A stochastic modeling and Monte Carlo simulation approach. Energy.

[7] Arzovs A., Judvaitis J., Nesenbergs K., Selavo L. (2024). Distributed learning in the iot–edge–cloud continuum. Mach. Learn. Knowl. Extr..

[8] Sincak P.J., Prada E., Miková L., Mykhailyshyn R., Varga M., Merva T., Virgala I. (2024). Sensing of continuum robots: A review. Sensors.

[9] Tu S., Li W., Zhang C., Wang L., Jin Z., Wang S. (2024). Seepage effect on progressive failure of shield tunnel face in granular soils by coupled continuum-discrete method. Comput. Geotech..

[10] Toscano J.D., Oommen V., Varghese A.J., Zou Z., Ahmadi Daryakenari N., Wu C., Karniadakis G.E. (2025). From pinns to pikans: Recent advances in physics-informed machine learning. Mach. Learn. Comput. Sci. Eng..

[11] Khalid S., Yazdani M.H., Azad M.M., Elahi M.U., Raouf I., Kim H.S. (2024). Advancements in Physics-Informed Neural Networks for Laminated Composites: A Comprehensive Review. Mathematics.

[12] Farea A., Yli-Harja O., Emmert-Streib F. (2024). Understanding physics-informed neural networks: Techniques, applications, trends, and challenges. AI.

[13] Hu H., Qi L., Chao X. (2024). Physics-informed Neural Networks (PINN) for computational solid mechanics: Numerical frameworks and applications. Thin-Walled Struct..

[14] Donnelly J., Daneshkhah A., Abolfathi S. (2024). Physics-informed neural networks as surrogate models of hydrodynamic simulators. Sci. Total Environ..

[15] Kapoor T., Wang H., Núñez A., Dollevoet R. (2024). Transfer learning for improved generalizability in causal physics-informed neural networks for beam simulations. Eng. Appl. Artif. Intell..

[16] Jalili D., Jadidi M., Keshmiri A., Chakraborty B., Georgoulas A., Mahmoudi Y. (2024). Transfer learning through physics-informed neural networks for bubble growth in superheated liquid domains. Int. J. Heat Mass Transf..

[17] Hussain A., Sakhaei A.H., Shafiee M. (2024). Machine learning-based constitutive modelling for material non-linearity: A review. Mech. Adv. Mater. Struct..

[18] Li Q.Q., Xu Z.D., Dong Y.R., He Z.H., Yan X., Wang B., Guo Y.Q. (2024). Characterization of dynamic mechanical properties of viscoelastic damper based on physics-constrained data-driven approach. Int. J. Struct. Stab. Dyn..

[19] Bergström J.S., Hayman D. (2016). An overview of mechanical properties and material modeling of polylactide (PLA) for medical applications. Ann. Biomed. Eng..

[20] Guo J., Wang H., Hou C. (2024). An adaptive energy-based sequential method for training PINNs to solve gradient flow equations. Appl. Math. Comput..

[21] Peng K., Li J. (2024). The coupled physical-informed neural networks for the two phase magnetohydrodynamic flows. Comput. Math. Appl..

[22] Guo J., Wang H., Gu S., Hou C. (2024). TCAS-PINN: Physics-informed neural networks with a novel temporal causality-based adaptive sampling method. Chin. Phys. B.

[23] Meng Q., Li Y., Liu X., Chen G., Hao X. (2023). A novel physics-informed neural operator for thermochemical curing analysis of carbon-fibre-reinforced thermosetting composites. Compos. Struct..

[24] Jiao A., Yan Q., Harlim J., Lu L. (2024). Solving forward and inverse PDE problems on unknown manifolds via physics-informed neural operators. arXiv.

[25] Rosofsky S.G., Al Majed H., Huerta E. (2023). Applications of physics informed neural operators. Mach. Learn. Sci. Technol..

[26] Kim T., Lee H., Lee W. Physics embedded neural network vehicle model and applications in risk-aware autonomous driving using latent features. Proceedings of the 2022 IEEE/RSJ International Conference on Intelligent Robots and Systems (IROS).

[27] Zhong Z., Ju Y., Gu J. Scalable Physics-Embedded Neural Networks for Real-Time Robotic Control in Embedded Systems. Proceedings of the 2024 IEEE 67th International Midwest Symposium on Circuits and Systems (MWSCAS).

[28] Li P., Ju S., Bai S., Zhao H., Zhang H. (2025). State of charge estimation for lithium-ion batteries based on physics-embedded neural network. J. Power Sources.

[29] Jia X., Willard J., Karpatne A., Read J.S., Zwart J.A., Steinbach M., Kumar V. (2021). Physics-guided machine learning for scientific discovery: An application in simulating lake temperature profiles. ACM/IMS Trans. Data Sci..

[30] Wang L., Zhu S.P., Luo C., Liao D., Wang Q. (2023). Physics-guided machine learning frameworks for fatigue life prediction of AM materials. Int. J. Fatigue.

[31] Chen J., Chen Y., Xu X., Zhou W., Huang G. (2022). A physics-guided machine learning for multifunctional wave control in active metabeams. Extreme Mechanics Letters.

[32] Ghaderi A., Dargazany R. (2023). A data-driven model to predict constitutive and failure behavior of elastomers considering the strain rate, temperature, and filler ratio. J. Appl. Mech..

[33] Ghaderi A., Ayoub G., Dargazany R. (2024). Constitutive behavior and failure prediction of crosslinked polymers exposed to concurrent fatigue and thermal aging: A reduced-order knowledge-driven machine-learned model. J. Mater. Sci..

[34] Ghaderi A., Chen Y., Dargazany R. (2022). A Physics-Based Data-Driven Approach for Modeling of Environmental Degradation in Elastomers. Proceedings of the ASME International Mechanical Engineering Congress and Exposition.

[35] Karniadakis G.E., Kevrekidis I.G., Lu L., Perdikaris P., Wang S., Yang L. (2021). Physics-informed machine learning. Nat. Rev. Phys..

[36] Zhang W., Ni P., Zhao M., Du X. (2024). A general method for solving differential equations of motion using physics-informed neural networks. Appl. Sci..

[37] Wu Y., Sicard B., Gadsden S.A. (2024). Physics-informed machine learning: A comprehensive review on applications in anomaly detection and condition monitoring. Expert Syst. Appl..

[38] Wang Y., Yao Y., Guo J., Gao Z. (2024). A practical PINN framework for multi-scale problems with multi-magnitude loss terms. J. Comput. Phys..

[39] Hashemi Z., Gholampour M., Wu M.C., Liu T.Y., Liang C.Y., Wang C.C. (2024). A physics-informed neural networks modeling with coupled fluid flow and heat transfer–Revisit of natural convection in cavity. Int. Commun. Heat Mass Transf..

[40] Seo J. (2024). Solving real-world optimization tasks using physics-informed neural computing. Sci. Rep..

[41] Jha N., Mallik E. (2024). GPINN with neural tangent kernel technique for nonlinear two point boundary value problems. Neural Process. Lett..

[42] Onyelowe K.C., Kontoni D.P.N. (2024). Numerical modeling of the funnel multiphysical flow of fresh self-compacting concrete considering proportionate heterogeneity of aggregates. Sci. Rep..

[43] Fang Z., Wang S., Perdikaris P. (2024). Learning only on boundaries: A physics-informed neural operator for solving parametric partial differential equations in complex geometries. Neural Comput..

[44] Stankovic D., Davidson J.R., Ott V., Bisby L.A., Terrasi G.P. (2024). Experimental and numerical investigations on the tensile response of pin-loaded carbon fibre reinforced polymer straps. Compos. Sci. Technol..

[45] Huang O., Saha S., Guo J., Liu W.K. (2023). An introduction to kernel and operator learning methods for homogenization by self-consistent clustering analysis. Comput. Mech..

[46] Jain A., Gurnani R., Rajan A., Qi H.J., Ramprasad R. (2025). A physics-enforced neural network to predict polymer melt viscosity. npj Comput. Mater..

[47] Tandia A., Onbasli M.C., Mauro J.C. (2019). Machine learning for glass modeling. Springer Handbook of Glass.

[48] Haywood-Alexander M., Liu W., Bacsa K., Lai Z., Chatzi E. (2024). Discussing the spectrum of physics-enhanced machine learning: A survey on structural mechanics applications. Data-Centric Eng..

[49] Qin B., Zhong Z. (2024). A Physics-Guided Machine Learning Model for Predicting Viscoelasticity of Solids at Large Deformation. Polymers.

[50] Zhang B. Intelligent Vehicle Lateral and Longitudinal Decoupled Dynamic Modeling and Control System Simulation Based on GRU-FNN. Proceedings of the 2024 3rd International Conference on Energy and Power Engineering, Control Engineering (EPECE).

[51] Ghaderi A., Morovati V., Dargazany R. (2020). A physics-informed assembly of feed-forward neural network engines to predict inelasticity in cross-linked polymers. Polymers.

[52] Ghaderi A. (2023). Physics-Informed Data-Driven Models for Inelastic, Aging, Failure Behavior of Crosslinked Polymers.

[53] Torzoni M., Rosafalco L., Manzoni A., Mariani S., Corigliano A. (2022). SHM under varying environmental conditions: An approach based on model order reduction and deep learning. Comput. Struct..

[54] Ghaderi A., Morovati V., Bahrololoumi A., Dargazany R. (2020). A physics-informed neural network constitutive model for cross-linked polymers. Proceedings of the ASME International Mechanical Engineering Congress and Exposition.

[55] Wang H., Bocchini P., Padgett J.E. (2024). Estimation of wind pressure field on low-rise buildings based on a novel conditional neural network. J. Wind. Eng. Ind. Aerodyn..

[56] Yang T., Li G., Li K., Li X., Han Q. (2024). The LPST-Net: A new deep interval health monitoring and prediction framework for bearing-rotor systems under complex operating conditions. Adv. Eng. Inform..

[57] Zhang L., Chen L., An F., Peng Z., Yang Y., Peng T., Song Y., Zhao Y. (2025). A physics-informed neural network for nonlinear deflection prediction of Ionic Polymer-Metal Composite based on Kolmogorov-Arnold networks. Eng. Appl. Artif. Intell..

[58] Jiang Q., Gou Z. (2025). Solutions to Two-and Three-Dimensional Incompressible Flow Fields Leveraging a Physics-Informed Deep Learning Framework and Kolmogorov–Arnold Networks. Int. J. Numer. Methods Fluids.

[59] Shuai H., Li F. (2025). Physics-informed kolmogorov-arnold networks for power system dynamics. IEEE Open Access J. Power Energy.

[60] Zhang S., Zhang C., Han X., Wang B. (2025). MRF-PINN: A multi-receptive-field convolutional physics-informed neural network for solving partial differential equations. Comput. Mech..

[61] Ryu Y., Shin S., Lee W.B., Na J. (2024). Multiphysics generalization in a polymerization reactor using physics-informed neural networks. Chem. Eng. Sci..

[62] Eivazi H., Tahani M., Schlatter P., Vinuesa R. (2022). Physics-informed neural networks for solving Reynolds-averaged Navier–Stokes equations. Phys. Fluids.

[63] Mahmoudabadbozchelou M., Karniadakis G.E., Jamali S. (2022). nn-PINNs: Non-Newtonian physics-informed neural networks for complex fluid modeling. Soft Matter.

[64] Singh P., Lalitha R., Mondal S. (2021). Saffman-Taylor instability in a radial Hele-Shaw cell for a shear-dependent rheological fluid. J. Non-Newton. Fluid Mech..

[65] Bian K., Priyadarshi R. (2024). Machine learning optimization techniques: A Survey, classification, challenges, and Future Research Issues. Arch. Comput. Methods Eng..

[66] Munir N., Nugent M., Whitaker D., McAfee M. (2021). Machine learning for process monitoring and control of hot-melt extrusion: Current state of the art and future directions. Pharmaceutics.

[67] Castillo M., Monroy R., Ahmad R. (2024). A cyber-physical production system for autonomous part quality control in polymer additive manufacturing material extrusion process. J. Intell. Manuf..

[68] Kasilingam S., Yang R., Singh S.K., Farahani M.A., Rai R., Wuest T. (2024). Physics-based and data-driven hybrid modeling in manufacturing: A review. Prod. Manuf. Res..

[69] Shi Y., Wei P., Feng K., Feng D.C., Beer M. (2025). A survey on machine learning approaches for uncertainty quantification of engineering systems. Mach. Learn. Comput. Sci. Eng..

[70] Soibam J., Aslanidou I., Kyprianidis K., Fdhila R.B. (2024). Inverse flow prediction using ensemble PINNs and uncertainty quantification. Int. J. Heat Mass Transf..

[71] Ju Y., Xu G., Gu J. 20.4 A 28nm Physics Computing Unit Supporting Emerging Physics-Informed Neural Network and Finite Element Method for Real-Time Scientific Computing on Edge Devices. Proceedings of the 2024 IEEE International Solid-State Circuits Conference (ISSCC).

[72] Kamath A.K., Anavatti S.G., Feroskhan M. (2024). A Physics-Informed Neural Network Approach to Augmented Dynamics Visual Servoing of Multirotors. IEEE Trans. Cybern..

[73] Farrag A., Kataoka J., Yoon S.W., Won D., Jin Y. (2024). SRP-PINN: A physics-informed neural network model for simulating thermal profile of soldering reflow process. IEEE Trans. Compon. Packag. Manuf. Technol..

[74] Xu Y., Zhang F., Zhai W., Cheng S., Li J., Wang Y. (2022). Unraveling of Advances in 3D-Printed Polymer-Based Bone Scaffolds. Polymers.

[75] Urraca R., Pernía-Espinoza A., Diaz I., Sanz-Garcia A. (2017). Practical methodology for validating constitutive models for the simulation of rubber compounds in extrusion processes. Int. J. Adv. Manuf. Technol..

[76] Wang G., Sun L., Zhang C. (2024). The effect of polyvinylpyrrolidone modified nano-polymers on rheological properties of silicon-based shear thickening fluid. Phys. Fluids.

[77] Zhu S., Wu S., Fu Y., Guo S. (2024). Prediction of particle-reinforced composite material properties based on an improved Halpin–Tsai model. AIP Adv..

[78] Jicsinszky L., Bucciol F., Chaji S., Cravotto G. (2023). Mechanochemical Degradation of Biopolymers. Molecules.

[79] Zhao Y., Xiao H., Chen L., Chen P., Lu Z., Tang C., Yao H. (2025). Application of the non-linear three-component model for simulating accelerated creep behavior of polymer-alloy geocell sheets. Geotext. Geomembr..

[80] Wu Z., Zhang H., Ye H., Zhang H., Zheng Y., Guo X. (2024). PINN enhanced extended multiscale finite element method for fast mechanical analysis of heterogeneous materials. Acta Mech..

[81] Liu D., Li Q., Zhu Y., Cheng R., Zeng T., Yang H., Yuan C. (2025). Physics-informed neural networks for phase-field simulation in designing high energy storage performance polymer nanocomposites. Appl. Phys. Lett..

[82] Qian F., Jia R., Cheng M., Chaudhary A., Melhi S., Mekkey S.D., Hu M. (2024). An overview of polylactic acid (PLA) nanocomposites for sensors. Adv. Compos. Hybrid Mater..

[83] Talwar D.N., Becla P. (2025). Microhardness, Young’s and Shear Modulus in Tetrahedrally Bonded Novel II-Oxides and III-Nitrides. Materials.

[84] Huang G., Zhang L., Chu S., Xie Y., Chen Y. (2024). A highly ductile carbon material made of triangle rings: A study of machine learning. Appl. Phys. Lett..

[85] Pateras J., Zhang C., Majumdar S., Pal A., Ghosh P. (2025). Physics-informed machine learning for automatic model reduction in chemical reaction networks. Sci. Rep..

[86] Ren D., Wang C., Wei X., Zhang Y., Han S., Xu W. (2025). Harmonizing physical and deep learning modeling: A computationally efficient and interpretable approach for property prediction. Scr. Mater..

[87] Proppe A.H., Lee K.L.K., Sun W., Krajewska C.J., Tye O., Bawendi M.G. (2025). Neural Ordinary Differential Equations for Forecasting and Accelerating Photon Correlation Spectroscopy. J. Phys. Chem. Lett..

[88] Gu Z.F., Yan Y.K., Wu S.F. (2025). Neural ODEs for holographic transport models without translation symmetry. Eur. Phys. J. C.

[89] Pyromali C., Taghipour H., Hawke L.G. (2024). Entangled linear polymers in fast shear: Evaluation of differential tube-based modeling including flow-induced disentanglement and chain tumbling. Rheol. Acta.

